# Personal Hygiene and Hot Weather

**Published:** 1905-07-15

**Authors:** 


### The Hospital.

A Journal of

The Medical Sciences and Hospital Administration.

Vol. XXXVIII.—No. 981. SATURDAY, JULY 15, 1905.

### Personal Hygiene and Hot Weather.

It is sometimes made a matter of complaint, by worthy people whose thoughts turn fondly towards the past, that we no longer have either the "old-fashioned" summers or the "old-fashioned" winters in which former generations were wont to delight, and from which they drew inexhaustible stores of health and energy. We hear, on the other hand, not merely of hot weather, but of "heat-waves," and there is literally no end to the eccentricities of the thermometers to which the editors of a certain class of newspapers appear to have access. We all remember the perplexities of the parish clerk immortalised by *Punch*, whose duty it was to warm the thermometer before the stove in anticipation of the coming of the curate; and some of the figures which we have seen in print seem to indicate that something of the same kind must be done prior to the arrival of the editor in more than one office situate in the locality of which the nominal centre is Fleet Street. These eccentricities apart, however, it is beyond question that on Saturday and Sunday last, the interior temperature of a carefully shaded room stood, for many hours together, at eighty degrees Fahrenheit; and the solar heat outside might without exaggeration be described as excessive, at least when contrasted with the temperatures to which we are mainly accustomed, and to which also our habits of life, our houses, and our clothing, have in the course of time been gradually adjusted. It is trite to remark that such periods of high temperature, although they occur almost every summer, find us always completely unprepared for them, and they are seldom of sufficient duration for it to be considered worth while, far less for it to be recognised as a necessity, that our business and family lives should in any degree be modified in the manner which longer spells of heat would imperatively require. Thousands of people wear the same dark and heavy clothes, eat the same superabundant meals, hurry from place to place in the same eager fashion and at the same hours of the day, whether the thermometer stands at 80° or at 60°. Their chief concession to the former temperature is to open their windows when they should be closed, and to encoura[illegible] abundant perspiration by copious draughts of fl[illegible] which is too often alcoholic in character. With their skins thus relaxed they ride outside omnibuses, or even on more rapidly moving tramcars, and thus at one and the same time chill the surfaces of their bodies and expose their heads and the backs of their necks to the full glare of the mid-day sun. Having done all these things, they complain of the heat, and not only compassionate themselves for having to endure it but marvel when they read of a few scattered cases of sunstroke.

There are plenty of instances in history of the custom of abandoning ordinary avocations in obedience to natural phenomena of occasional occurrence. Every one of Archbishop Temple's old schoolfellows at Tiverton, or, for the matter of that, every reader of "Lorna Doone," will remember that the work of Blundell's school was interrupted, and that the day-boys were sent home, as soon as the water of the river Lowman in flood reached the initials of the founder in the pavement at the outer gate; and, at least to some people, solar heat in excess is more dangerous than an inundation. If we lived in a country where the thermometer reached 80° during three or four months of the year, it is quite certain that we should modify our habits accordingly; and there can be no valid reason why we should not do so under the sway either of enlightened legislation or of reasonable custom during the shorter and uncertain period of the annual recurrence of such a temperature. When a standard thermometer reached a certain point, earlier hours and midday siestas should be the rule.

In the absence of such rule, it is still possible for individuals to protect themselves against excessive heat in a far greater degree than is now customary. Even for people of very small means, the provision of a suit of thin and light-coloured clothing, to be reserved for periods of high temperature, would not involve any excessive outlay; and the same may be said of the purchase of a white umbrella, loosely lined with some darker material, and utilised to protect the head and spine from the direct rays of the sun. Again, an allowance of five or ten minutes beyond the ordinary time for the daily journey to business would eliminate one of the great sources of hurry in commercial life. At home, windows should be shut and blinds lowered before the sun falls upon them, and the direct rays, whether luminous or calorific, excluded as much as circumstances will allow. At night, of course, the same windows should be widely open, especially in sleeping rooms, or wherever they can so be left without danger from thieves—a danger which may usually be obviated by properly placed screw fastenings. Among the labouring classes thousands of families close their windows from sheer love of foul air, and in many suburbs there is much temptation to do so on account of the noise of the nocturnal traffic. Noise, however, is something which soon ceases to disturb a sleeper, and even broken rest is better than asphyxiation. Last, but not least, there is the question of hot-weather diet. If the ordinary allowance of flesh food were cut down to one-third and that of starch and vegetables to one-half, a great step in the direction of health and comfort would be taken. And were the usual daily average of fluid not exceeded the excessive perspiration with its attendant discomforts would be avoided and no excessive thirst would be experienced. Much drinking is an evil which grows by what it feeds on, and when heat is productive of a feeling of real exhaustion, a single mouthful of iced water is usually the most effectual remedy.

